# New insights in the relative radiobiological effectiveness of proton irradiation

**DOI:** 10.1186/s13014-018-0954-9

**Published:** 2018-01-16

**Authors:** K. Ilicic, S. E. Combs, T. E. Schmid

**Affiliations:** 1 Department of Radiation Oncology, Klinikum rechts der Isar, Technische Universität München, 81675 München, Germany; 2 Institute of Innovative Radiotherapy, Helmholtz Zentrum München, Neuherberg, Germany; 3 Deutsches Konsortium für Translationale Krebsforschung (DKTK), Partner Site Munich, Munich, Germany

**Keywords:** Proton, Radiotherapy, RBE, Bragg peak

## Abstract

**Background:**

Proton radiotherapy is a form of charged particle therapy that is preferentially applied for the treatment of tumors positioned near to critical structures due to their physical characteristics, showing an inverted depth-dose profile. The sparing of normal tissue has additional advantages in the treatment of pediatric patients, in whom the risk of secondary cancers and late morbidity is significantly higher. Up to date, a fixed relative biological effectiveness (RBE) of 1.1 is commonly implemented in treatment planning systems with protons in order to correct the physical dose. This value of 1.1 comes from averaging the results of numerous in vitro experiments, mostly conducted in the middle of the spread-out Bragg peak, where RBE is relatively constant. However, the use of a constant RBE value disregards the experimental evidence which clearly demonstrates complex RBE dependency on dose, cell- or tissue type, linear energy transfer and biological endpoints. In recent years, several in vitro studies indicate variations in RBE of protons which translate to an uncertainty in the biological effective dose delivery to the patient. Particularly for regions surrounding the Bragg peak, the more localized pattern of energy deposition leads to more complex DNA lesions. These RBE variations of protons bring the validity of using a constant RBE into question.

**Main body:**

This review analyzes how RBE depends on the dose, different biological endpoints and physical properties. Further, this review gives an overview of the new insights based on findings made during the last years investigating the variation of RBE with depth in the spread out Bragg peak and the underlying differences in radiation response on the molecular and cellular levels between proton and photon irradiation. Research groups such as the Klinische Forschergruppe Schwerionentherapie funded by the German Research Foundation (DFG, KFO 214) have included work on this topic and the present manuscript highlights parts of the preclinical work and summarizes the research activities in this context.

**Short conclusion:**

In summary, there is an urgent need for more coordinated in vitro and in vivo experiments that concentrate on a realistic dose range of in clinically relevant tissues like lung or spinal cord.

## Background

Today, more than 50% of all cancer patients are treated with radiotherapy [1], mostly with high-energy X-rays, which are produced by linear accelerators [2]. Charged particle beams such as protons offer many advantages compared to the radiotherapy with X- rays due to a fundamental difference between the physical properties. Proton therapy is one of the newer radiation treatment modalities and in contrast to the conventional radiotherapy with X-rays, proton beams can be deposited in precise areas with minimal lateral scattering in tissue, which reduces the irradiation to the healthy tissue surrounding the tumor providing reduced side effects [2–4]. Due to their physical properties protons are preferentially applied in the treatment of tumors located near to critical structures such as spinal cord, eyes and brain as well as in pediatric malignancies [5]. Relative biological effectiveness (RBE) is a value used to account for differences in radiobiological effect between photons and other particles employed for radiation treatments. For clinical patient treatment, a constant relative biological effectiveness (RBE) of 1.1 is currently recommended and applied for proton beams [6, 7], despite the fact that the RBE of protons depends on many factors such as dose level, linear-energy transfer (LET), tissue radio-sensitivity, oxygen concentration and biological end-points. This equivalence to photon irradiation has been mainly driven by the lack of clinical data to suggest any significant difference. This uncertainty in the RBE translates to an uncertainty in the biological effective dose delivered to a patient. Given that proton radiation induces only a 10% higher RBE when compared to conventional photon therapy, it has been generally accepted that proton therapy is unlikely to improve overall patient survival. Preliminary evidence from non-randomized clinical studies has shown that proton therapy provides better local control in NSCLC and meningioma [8, 9], however this has to be confirmed in randomized studies.

However, in the last years, there is a growing body of evidence suggesting that particularly near the edges of the high-dose volume, the fall-off portion of the Bragg peak, the RBE of protons is significantly higher. Several in vitro studies investigating different points along a proton beam suggested a significantly higher RBE [10–12]. Recent modeling studies suggest that there are significant differences between the biologically weighted dose and the absorbed dose distributions for both tumor and normal tissues [13]. Due to the recent findings, the accuracy of a fixed RBE value is being questioned with respect to its efficacy and safety. Therefore, this review analyzes the relationships of the RBE with dose, biological endpoint and the physical properties.

### Radiobiology of protons

Clinical practice assumes a fixed proton RBE of 1.1, but it has been postulated that higher RBEs occur at the distal edge of proton spread out Bragg peak (SOBP). However, apart from the advantages offered by depth-dose profile of protons, they also show an enhanced biological effectiveness in cell killing [7]. This is related to the increased LET compared to X-rays when protons are close to the Bragg peak. Therefore, the use of ion beams for radiation therapy is currently undergoing investigation at different institutions. In 2008 a meeting on RBE in ion beam therapy dealt primarily with a review of experimental measuring of RBE and approaches to the clinical use of the concept of RBE based on experimental findings, theoretical models and previous clinical experience with protons and heavy ions [14]. Although the physical aspects of proton beam radiobiology are well understood, the biological aspects, particularly the complex biological endpoints need further attention. The current estimates of RBE depend on the cell type and also on the detection methods because it has been shown that DNA damage and apoptotic responses vary greatly between gamma radiation and proton therapy in a tissue- and dose-dependent fashion [15]. Experimental data emerging from recent studies suggest that, for several endpoints of clinical relevance, the biological response is differentially modulated by protons compared to photons. However, up to date only few studies have been performed to understand the differential response on the molecular and cellular levels between proton and photon irradiation. Several studies reported an increased induction of double strand breaks (DSBs) and more complex DNA damage induced by protons in comparison to photon irradiation [16, 17]. DNA DSB induction by different radiation qualities shows that, even though similar patterns of initial induced DSBs are produced by photons and protons, there are differences when looking at the rejoining process [18]. Another study demonstrated that lesions induced by proton irradiation were preferentially repaired by homologous recombination, a much slower repair mechanism than Non-Homologous End Joining, which could be attributed to the increased complexity after proton irradiation [19]. This also affects the number of residual lesions measured late after irradiation. Another study found differences between photon and proton irradiation reactive oxygen species dependent mechanism by which proton radiation induces DNA damage and cell apoptosis [4]. In the study of Di Pietro et al., lower percentage of apoptotic cells was found after photon irradiation and apoptosis was induced in a temporally delayed fashion compared to protons [20]. The study of Manti et al., showed increased amounts of complex chromosomal aberrations as well as increased frequency of sister chromatid exchanges after proton irradiation [21]. The study of Green et al., found that micronuclei formation and apoptosis induction were higher in thyroid follicular cells after proton irradiation compared to photon irradiation [22]. Also different epigenetic changes where reported after proton and photon irradiation. Exposure to X-rays was associated with hypo-methylation, while proton irradiation produced mainly hyper-methylated DNA, both in normal and cancer cells [23]. For the gold standard on the cellular level, the colony formation assay, many in vitro studies were published up to now. Using the colony formation assay an average RBE of 1.1–1.2 can be associated to the middle of the SOBP [6, 7, 24, 25]. A lower level of migration and a reduced invasion potential has been reported after proton irradiation in comparison to X-rays [11]. Interestingly, protons show anti-invasive and anti-migration behavior. The studies of Girdhani et al., showed lower levels of migration and invasion after proton irradiation in comparison to X-rays [26, 27]. Unfortunately, there are still no randomized trials available for second cancer induction in patients treated with proton vs. photon radiation. There are only very few studies which suggest that the rate of second cancer induction is less than 50% after proton irradiation compared to photon radiation [28].

### The relationship between LET and RBE

In recent years, modeling of RBE as a function of LET receives much attention in the proton therapy community [29]. However, these LET-RBE parametrizations are ion type specific and their application is restricted by large uncertainties associated with the biological input parameters from proton experiments [29]. The RBE is defined as the ratio of a dose of sparsely ionizing radiation, mostly photons to a dose of any other radiation quality to produce the same biological effect. High LET radiation has an increased biological effectiveness compared to photons of low LET. Carbon or oxygen ions offer a higher RBE due to the severe radiation damage produced within the beam track. However, data on in-vitro RBE evaluation of high-LET irradiations are still sparse. Recently, our group reported RBE-datasets for carbon and oxygen ion and examined the effect of additional anti-tumorigenic substances [30–33]. The main reason for an increased biological effectiveness is the clustered damages to the DNA structure within one nucleus, which is more difficult for the cell to repair and which leads to increased cell killing [34]. As a result, the RBE varies spatially within the patient and increases toward the distal end of a SOBP, as LET values increases with the depth of the beam [35]. It is known that the RBE is highly dependent on both cell type and the studied endpoint but also on particle species, due to the different dose deposition profiles on microscopic scale [36]. The study of Rorvik et al., developed linear as well as and non-linear RBE models for protons by applying the LET spectrum as a parameter for the radiation quality [35]. The study demonstrated that non-linear models give a better representation of the RBE-LET relationship for protons compared to linear models. Therefore, the LET is not sufficient as a predicting factor of RBE. In general, the RBE depends on the microdose distribution formed by a single ion track and the areal ion track density determining the total dose. Due to the complex RBE dependency, biophysical models are essential for the estimation of clinically relevant RBE values in treatment planning [37]. There are some approaches to model radiobiological endpoints based directly on the microdose distribution [38–40] the three-dimensional dose distribution with nanometer resolution deposited by a single particle. An important biophysical prediction model that is currently implemented in the treatment panning systems for the heavy ion radiotherapy in Europe is the local effect model (LEM) [37, 41]. This model is used to predict the RBE for cell killing in order to correct the physical dose required for the tumor irradiation with heavy ions. According to the latest version of the LEM (LEM IV) [42, 43] the spatial DNA DSB distribution and their local density within a cell nucleus are assumed to be the most relevant factors that influence the cell fate following radiation.

**Fig. 1:**
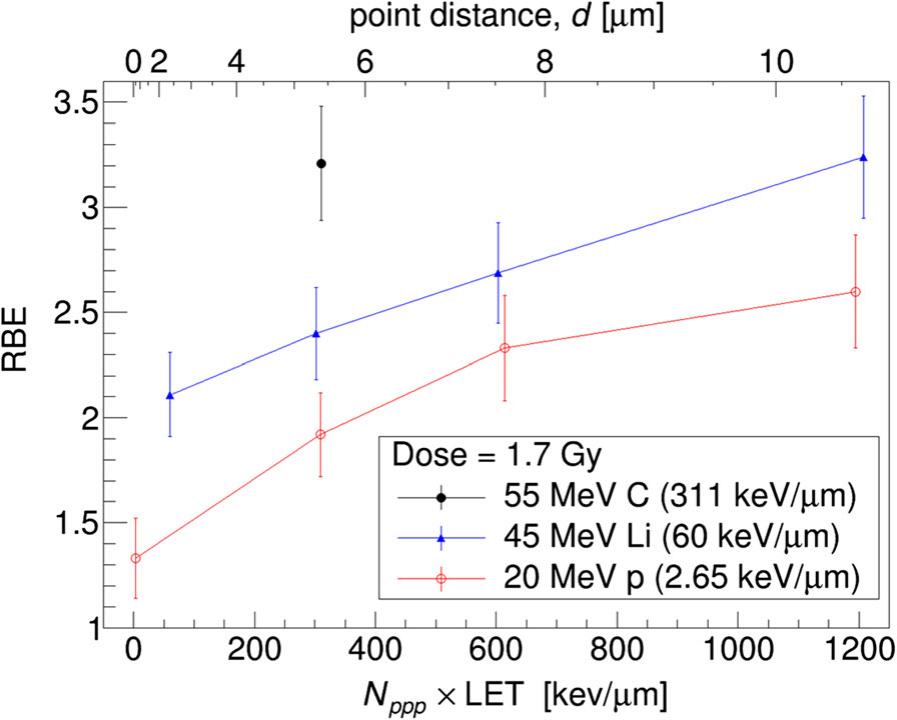
20 MeV protons versus the number of particles per point multiplied by the LET

It is known that the energy deposition for high LET radiation is much more inhomogeneous in time and space than that of low LET radiation [44]. The energy deposition of a single ion hit into a biological cell runs on the femtosecond to picoseconds time scale, while the spatial dose distribution peaks at the center of the ion track [45]. It was shown already in the 70ies and 80ies of the last century that spatial distributions of energy deposition events and the resulting DSB distributions do affect the outcome as shown using spatially correlated ions which were produced from diatomic ions [46, 47]. Recently, the influence of spatial dose distribution on the RBE with respect to different biological endpoints has been investigated using an experimental approach where low LET 20 MeV protons (LET = 2.65 keV/m) were focused to sub-micrometer spots in cell nuclei [44, 45, 48]. Here, the authors reported on an enhanced RBE with regard to induction of dicentric chromosomes and micronuclei in hybrid human-hamster A_L_ cells after spot application of a bunch of 20 MeV protons compared to a quasi-homogeneous irradiation [45]. In another manuscript A_L_ cells have been irradiated with 20 MeV (2.6 keV/m) protons quasi-homogeneously distributed or focused to 0.5 × 1 μm^2^ spots on regular matrix patterns (point distances up to 10.6 × 10.6 μm), with pre-defined particle numbers per spot to provide the same mean dose of 1.7 Gy [44]. The yields of dicentrics and their distribution among cells have been scored. The yields of dicentric chromosomes increased by focusing up to a factor of 2 for protons compared to quasi-homogeneous irradiation (Fig. 1). The local density of DNA DSBs increased at the irradiated spots enhancing also the probability for the interaction of the DSBs and thus increasing the probability of connecting the wrong ends. The reported study improved the understanding of the mechanisms by which radiation induces these lethal chromosome aberrations [44].

Furthermore, variation of the spatial DSB distribution within a cell nucleus by focusing low LET protons resulted in a higher cell killing compared to quasi homogeneous proton application [48]. These results indicate that the sub-micrometer proton focusing, which affects the DSB distribution within the cell nucleus leads to decreased cell survival [44, 48]. Thus significant variations in RBE can be expected if low LET protons are applied in a spatially correlated manner. Moreover, these results strongly support the assumption of the LEM model that the spatial DNA damage distribution is the source of relative biological effectiveness [45].

### Variation of RBE along the SOBP

In recent years, the fixed RBE value of 1.1 is being questioned with respect to its safety, because if the dose to the tumor is too low, the risk of tumor recurrence increases. On the other hand, if the dose is too high, the chances for acute and last side effects will increase. Disregarding this RBE and LET variations could have negative clinical implications, especially when an organ at risk is located near the distal end of a tumor [35]. A fixed RBE during fractionated exposures disregards any effects due to the variation of dose per fraction and the total number of fractions delivered in relation to the LET. However, a number of recent in vitro studies have reported that the RBE within the SOBP is not constant and the RBE increases at the distal end of the SOBP. Table 1 summarizes these in vitro studies. The study of Britten et al., demonstrated that the RBE of the proton beam at certain depths is greater than 1.1 and therefore there is an increased potential for cell killing and normal tissue damage in the distal regions of the Bragg peak [10]. Proton beam therapy has a higher LET rate, particularly toward the distal edge of the SOBP, compared with conventional X-ray radiation. An enhanced efficiency in the induction of cell inactivation can be measured at different positions along the SOBP [49, 50]. Differences in the RBE which are depending on the position along the SOBP were reported in several studies. The study of Petrovic et al., found an increased killing ability at the SOBP distal edge, which was the consequence of increasing proton LET [51]. Another study reported on the variation of the RBE with depth in the SOBP of the 76 MeV proton beams, where they found that, despite a homogeneous physical dose, the tumor cells at the distal end receives a higher biologically equivalent dose than at the proximal end [16]. More recent, the study of Hojo et al., demonstrated that the RBE using an high-energy proton beam, differed according to the position on the SOBP in two human esophageal cancer cell lines with differing radiosensitivities [52]. Also the number of unrepaired double-stranded DNA breaks, as assessed by the number of γ-H2AX foci assay 24 h after irradiation was higher for irradiation at the distal end of the SOBP. In a theoretical study of Carante and Ballarini, a biophysical model of radiation-induced cell death and chromosome aberrations called Biophysical Analysis of Cell death and chromosome Aberrations (BIANCA) was used in order to predict the cell death and the yield of dicentric chromosomes at different depth positions along a SOBP dose profile of therapeutic protons [53]. These simulation data are consistent with the experimental cell survival data as reported in Chaudhary et al. [11] and for both investigating endpoints an increased beam effectiveness was shown along the plateau, implying that the assumption of a constant RBE along a proton SOBP may be sub-optimal [53]. The results of an ex vivo study, where the intestine of mice was irradiated with 200 MeV clinical proton beam are consistent with in vitro data showing an increased proton RBE with depth in an SOBP for both investigated biological endpoints, the intestinal crypt regeneration and lethal dose 50% (LD_50_) [54]. The study of Marshall et al. have analyzed clinical implications of a variable RBE on proton dose fractionation in human skin fibroblast (AG01522) cells using pencil scanned proton clinical beam of maximum energy 219.65 MeV. Their findings have shown significant variations in the cell killing RBE for both acute and fractionated exposures along the proton dose profile, with a sharp increase in RBE toward the distal position [55]. The study of Chaudhary et al. used the same cell line and investigated the DNA damage response after irradiation with a modulated SOBP and a pristine proton beam, as this new delivery technique was applied in form of intensity-modulated particle therapy (IMPT) in more and more proton therapy centers worldwide [56]. A significantly higher frequency of persistent DNA damage foci was observed at the distal end of the SOBP, whereas the irradiation with a monoenergetic proton beam resulted in significantly increased number of foci at Bragg peak position 24 h after irradiation [56]. In the study of Guan et al. clonogenic cell survival has been mapped as a function of LET along pristine scanned proton beam and the findings indicated that the measured biological effects are greater than reported in previous studies [57]. Furthermore a non-linear RBE for cell survival as a function of LET near and beyond the Bragg peak was observed in this study.

**Table 1:** RBE versus SOBP

Reference	Biological system	Biological endpoint	Beam [MeV]	SOBP [cm]	RBE (position of SOBP)	Ref. radiation
Calugaru et al., 2011 [16]	Human cervix cancer cells HeLa/Head and neck squamous cancer cells SQ20B	Cell survival SF = 0.37	76 201	3 20	1.07/1.09 (entrance), 1.14/1.17 (mid-SOBP), 1.33/1.30 (distal) No variation with depth along SOBP for 201-MeV energy beam	^137^Cs γ-rays
Wouters et al., 2015 [24]	Chinese hamster cells V-79	Cell survival A) SF = 0.34 B) SF = 0.71	160 230	10	A) 1.07 (entrance), 1.10 (prox. half), 1.17 (distal half) and 1.21 (distal edge) Similar effects also for 230 MeV beam B) 1.13 (entrance), 1.15 (prox. half), 1.26 (distal half), 1.30 (distal edge)	^60^Co γ-rays
Cuaron et al., 2016 [12]	U2OS	DNA damage repair A) 3 h B) 24 h	152	10	RBE increases as a function of depth along the Bragg peak A) RBE > 2 (entrance), RBE > 4.0 (distal) B) RBE > 2 (entrance), RBE > 6.0 (distal)	6 MV X-rays
Britten et al., 2013 [10]	Human laryngeal cancer cells Hep2/ Chinese hamster cells V79	Cell survival SF = 0.10	87 200		1.46/1.23 (mid), 2.1/1.46 (distal), 2.3/1.78 (dose fall-off) Similar D_0.1_ isoeffect RBE values as for 200 MeV proton beam irradiation	^60^Co γ-rays
Chaudhary et al., 2014 [11]	Human fibroblasts AG01522 and glioma cells U87	Cell survival	62		RBE increases for both cell lines and SF = 0.50, SF = 0.10 and SF = 0.01 as a function of depth of the SOBP	225 kVp X-rays
Matsumoto et al., 2014 [13]	Human salivary gland tumor cells HSG	Cell survival A) SF = 0.10 B) SF = 0.60	190	5	A) 1.24 (150 mm- middle), 1.5 (180 mm - distal) B) 1.20 (150 mm- middle), 1.86 (180 mm - distal)	6 MV X-rays
Bettega et al., 2000 [50]	Human squamous cell carcinoma of the tongue SCC25	Cell survival SF = 0.10	65		0.99 (2 mm) – entrance 1.04 (15.6 mm) and 1.22 (25 mm) – in the SOBP 1.34 (27.2 mm) and 1.98 (27.8 mm) – distal declining edge	^60^Co γ-rays
Petrovic et al., 2010 [51]	HTB140 melanoma	Cell survival	62		1.68–2.84 at the distal end of SOBP 7.14 at its distal declining edge	Middle of the SOBP
Hojo et al., 2017 [52]	Human esophageal cancer cell lines OE21/KYSE450	Cell survival A) SF = 0.10 B) SF = 0.37	235		A) 1.06/1.03 (entrance), 1.17/1.06 (proximal), 1.22/1.20 (middle), 1.24/1.24 (distal) B) 1.16/1.02 (entrance), 1.33/1.09 (proximal), 1.31/1.21 (middle), 1.40/1.27 (distal)	6 MV X-rays
Slabbert et al., 2015 [54]	Ex vivo murine jejunum	Regeneration of intestinal crypts	200	A) 3 B) 7	A) RBE increase of 5% ± 3% from the middle to the intermediate position, and an RBE increase of 9% ± 4% from the middle to the end of the SOBP B) RBE increase of 10% ± 4% from the middle to the end of the SOBP	^60^Co γ-rays
Marshall et al., 2016 [55]	Human skin fibroblasts AG01522	Cell survival as a function of total dose delivered in a single (A) and triple exposure (B) SF = 0.10	219.65		A: 1.02 (entrance), 1.13 (proximal), 1.25 (center), 1.40 (distal) B: 1.11 (entrance), 1.31 (proximal), 1.40 (center), 2.01 (distal)	225 kVp X-rays
Chaudhary et al. 2016 [56]	Human skin fibroblasts AG01522	DNA damage repair	60	Modulated SOBP and monoenergetic proton beam	Modulated SOBP: increased complexity of DNA lesions at the distal end of SOBP and slower repair kinetics Pristine beam: Significantly increased number of foci at Bragg peak position 24 h after irradiation	225 kVp X-rays
Guan et al., 2015 [57]	Non-small cell lung cancer cells H460 and H1437 (p53 mutant)	Cell survival SF = 0.10	79.7	Monoenergetic scanning beam with 4.8 cm range in water	Increased RBE at and beyond the Bragg peak. Non-linear relationship between RBE and LET for both cell lines, RBE scaled in a biphasic maner	^137^Cs γ-rays

It is important to note, that the RBE predicted by the LEM is in better agreement with the experimental data within the SOBP region than with the constant RBE of 1.1 that is currently applied in the clinics [58]. However, the LEM predictions and experimental data show only a weak dependence of RBE on the tissue type, which is considered insignificant with regard to the general uncertainties of RBE [58].

Recently, clinical evidence for variations in proton RBE was demonstrated by the study of Peeler et al., where the authors analyzed correlation of the tissue damage with increased biological dose effectiveness in pediatric ependymoma patients after proton therapy [59]. Their findings have shown that voxel-based changes on post-treatment MR images are associated with increased LET and dose.

## Conclusion

Up to date, radiotherapy using protons are currently planned using the assumption that the proton RBE relative to photons is 1.1. However, this assumption ignores the experimental evidence which clearly demonstrates that proton RBE varies along the treatment field with LET.

In this review the latest studies which showed that the RBE varies within the SOBP have been summarized. Accordingly, experimental in vitro data indicate that the highest RBE within the SOBP is found at the distal edge and in the distal fall-off region. The latest findings help clarify the underlying differences in radiation response on the molecular and cellular levels between proton and photon irradiation. This increase in RBE as a function of depth results in an extension of the bio-effective range of proton the beam in patients. Further, because RBE values may increase with deceasing dose causing elevated RBE values for organs at risk compared to the target area. In order to incorporate detailed RBE modeling the assumption of the LEM model that the spatial DNA damage distribution is the source of relative biological effectiveness should be considered. However, despite the recent studies, more efforts are urgently needed to increase the accuracy of the evaluation of RBE for proton radiotherapy. Current experiments in normal and tumor tissue along the SOBP, are well justified and should be continued.

Even though the current in vitro data so far indicate a suboptimal application of a generic RBE of 1.1 these are not sufficient to change the clinical use of a constant RBE. Particularly, better knowledge and understanding of protons RBE variations are necessary in vivo, before RBE variations can be implemented in proton radiotherapy. Therefore preclinical and clinical studies are urgently needed to clarify how the inhomogeneity of the RBE within the range of the SOBP would affect the clinical outcomes.

## Abbreviations

DSB: Double strand break
LET: Linear energy transfer
RBE: Relative biological effectiveness
SOBP: Spread out bragg peak
